# The Liver Transection Area Is a Novel Predictor for Surgical Difficulty in Laparoscopic Liver Resection

**DOI:** 10.3390/jcm13195686

**Published:** 2024-09-24

**Authors:** Motohiko Yamada, Kosei Takagi, Tomokazu Fuji, Kazuya Yasui, Jiro Kimura, Takeyoshi Nishiyama, Yasuo Nagai, Noriyuki Kanehira, Toshiyoshi Fujiwara

**Affiliations:** Department of Gastroenterological Surgery, Okayama University Graduate School of Medicine, Dentistry, and Pharmaceutical Sciences, Okayama 700-8558, Japan; me17090@s.okayama-u.ac.jp (M.Y.); pri958hs@s.okayama-u.ac.jp (T.F.); pjyv6nvp@s.okayama-u.ac.jp (K.Y.); me17022@s.okayama-u.ac.jp (J.K.); me17063@s.okayama-u.ac.jp (T.N.); pfqz3zaq@s.okayama-u.ac.jp (Y.N.); p63m7e48@s.okayama-u.ac.jp (N.K.); toshi_f@md.okayama-u.ac.jp (T.F.)

**Keywords:** laparoscopic liver resection, surgical difficulty, liver transection area

## Abstract

**Background**: A difficulty scoring system was developed to estimate the surgical outcomes of laparoscopic liver surgery (LLS); however, the effect of the liver transection area (LTA) on LLS outcomes have not been previously examined. Therefore, this study investigated the predictive significance of the LTA for LLS. **Methods**: This retrospective study included 106 patients who underwent LLS in our hospital between January 2012 and December 2023. The association of the LTA with the surgical difficulty level and operative time was investigated. Multivariate analyses were performed to identify factors predicting surgical difficulty in LLS. **Results**: The median LTA and operative time were 62.5 (IQR, 36.0–91.8) cm^2^ and 250 (IQR, 195–310) minutes, respectively. The LTA was significantly associated with surgical difficulty as evaluated using the IWATE Criteria. Moreover, the LTA significantly correlated with operative time (r^2^ = 0.19, *p* < 0.001). The multivariable analyses found that the LTA (≥59 cm^2^) (odds ratio [OR], 6.07; 95% confidence interval [CI], 2.38–16.6; *p* < 0.001) and the type of LLS (≥segmentectomy) (OR, 3.79; 95% CI, 1.35–11.4; *p* = 0.01) were significant factors associated with surgical difficulty. **Conclusions**: The LTA is a useful parameter that reflects the difficulty of LLS.

## 1. Introduction

The advantages of minimally invasive liver resection over open surgery have been well established, including lower blood loss and complications, faster functional recovery, a shorter hospital stay, and similar oncological outcomes [1,2]. Although laparoscopic liver surgery (LLS) has become a standard procedure, technically complex LLS, including hemihepatectomy and the resection of the posterosuperior segments, should be safely performed by experienced surgeons [3]. Therefore, it is important for surgeons to understand surgical difficulties and gradually improve their surgical skills for the safe implementation of LLS [4].

Initially, a difficulty scoring system for LLS was developed in 2014 that was based on the extent of liver resection, tumor location, tumor size, liver function, and tumor proximity to major vessels [5]. Subsequently, a difficulty scoring system (IWATE Criteria) was modified to estimate surgical outcomes in LLS [6]. The scoring system included six parameters, such as the tumor location, tumor size, extent of liver resection, and liver function. Although the effectiveness of the existing difficulty scoring systems has been investigated [7], other factors may influence the surgical difficulty associated with LLS. In clinical practice, the difficulty of LLS differs depending on tumor depth and diameter, even in partial liver resection [8]. We hypothesized that the liver transection area (LTA) may influence the difficulty of performing LLS. Overall, this study aimed to investigate the predictive significance of the LTA for LLS.

## 2. Materials and Methods

### 2.1. Patients

This retrospective study was performed using medical records of 167 consecutive patients who underwent LLS at our institution between January 2012 and December 2023. Patients who underwent hand-assisted LLS, repeat LLS, or conversion surgery were also excluded. This study was approved by the ethics committee of our institution and conducted in accordance with the Declaration of Helsinki. The need for informed consent was waived due to the retrospective nature of the study.

### 2.2. Clinical Data

The following data were extracted from the database: age, sex, body mass index, American Society of Anesthesiologists (ASA) physical status [9], comorbidity (diabetes, hypertension, and hepatitis B virus and hepatitis C virus infections), previous abdominal surgery, laboratory values, liver function (indocyanine green retention 15 min and the Child–Pugh score), etiology of liver disease (hepatocellular carcinoma, intrahepatic cholangiocarcinoma, metastatic tumor, and benign tumor), tumor factors (size, number, and location), type of hepatectomy, operative time, blood loss, and postoperative outcomes (mortality, major complication evaluated by the Clavien–Dindo grade ≥ 3 [10], and hospital stay).

The surgical difficulty levels of LLS were evaluated using the IWATE Criteria (a 4-level classification system involving 6 preoperative factors): low (1–3), intermediate (4–6), advanced (7–9), and expert (10–12) [6]. The investigated variables included the resection extent, tumor location, tumor size, liver function, proximity to major vessels, and hand-assisted/hybrid IWATE Criteria [6].

### 2.3. Liver Transection Area Measurement

**Figure 1:**
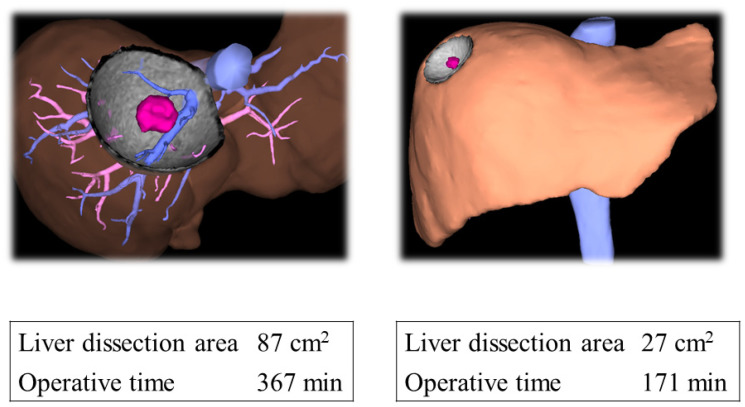
The liver transection area measurement for laparoscopic segment 8 resection. Even in the same segment resection, the surgical difficulty will differ depending on the liver transection area (LTA). Specifically, a larger LTA leads to longer operative times.

The LTA (cm^2^) was calculated using computed tomography (CT) image analysis system (Synapse Vincent; Fujifilm Medical, Tokyo, Japan) (Figure 1). Postoperatively, we confirmed that all surgical liver transection lines were consistent with those predicted preoperatively using the Vincent system.

### 2.4. Surgical Technique

The surgical indications were determined through multidisciplinary meetings. The extent of liver resection was determined by considering oncological factors and liver function. A pneumoperitoneum was established with an average pressure of 10 mmHg. The liver parenchyma was transected using a Cavitron Ultrasonic Surgical Aspirator (CUSA) and ultrasonic shears (Ligasure) [11]. The intermittent Pringle maneuver was used for inflow control with a tourniquet system.

### 2.5. Statistical Analysis

Initially, we investigated the relationship between the LTA and difficulty level using the IWATE Criteria [6]. Subsequently, the cutoff value of LTA associated with a prolonged operative time (>250 min) was examined using a receiver operating characteristic curve. An operative time longer than the median operative time was defined as prolonged operative time in this study. Finally, univariate and multivariate logistic regression analyses were performed to identify factors predicting surgical difficulty (prolonged operative time). Based on the results of the multivariate analyses, internal validity was evaluated using the bootstrap method to assess the discriminative performance of the model [12]. The calibration curve C-index was used to determine the predictive validity of the model. Odds ratios (ORs) and 95% confidence intervals (CIs) were determined. All statistical analyses were performed using the JMP software (version 11; SAS Institute) and EZR software (version 1.65; Saitama Medical Center, Jichi Medical University, Saitama, Japan).

## 3. Results

### 3.1. Patient Characteristics

Of 167 patients, 106 were included after excluding those who underwent hand-assisted LLS (*n* = 21), repeat LLS (*n* = 17), conversion surgery (*n* = 6), or other procedures (*n* = 17). The characteristics of the 106 patients are presented in Table 1. The cohort included 63 men and 43 women with a median age of 68 years (IQR, 59–73 years). Common primary diseases included hepatocellular carcinoma (*n* = 45) and metastatic liver tumors (*n* = 47). All patients had Child–Pugh score A with a median indocyanine green retention 15 min level of 9.4 (IQR, 5.9–14.1). The difficulty levels were classified as low (*n* = 30), intermediate (*n* = 51), advanced (*n* = 18), or expert (*n* = 7). The median LTA was 62.5 (IQR, 36.0–91.8) cm^2^.

The hepatectomy types included partial resection (*n* = 54), left lateral sectionectomy (*n* = 19), segmentectomy (*n* = 3), sectionectomy (*n* = 18), and hemihepatectomy (*n* = 12). The operative time and estimated blood loss were 250 min (IQR, 195–310) and 65 mL (IQR, 10–170), respectively. In this study, the incidence rates of mortality and major complications were 0% and 3.8%, respectively.

### 3.2. Association between Liver Transection Area and Difficulty Level

**Figure 2:**
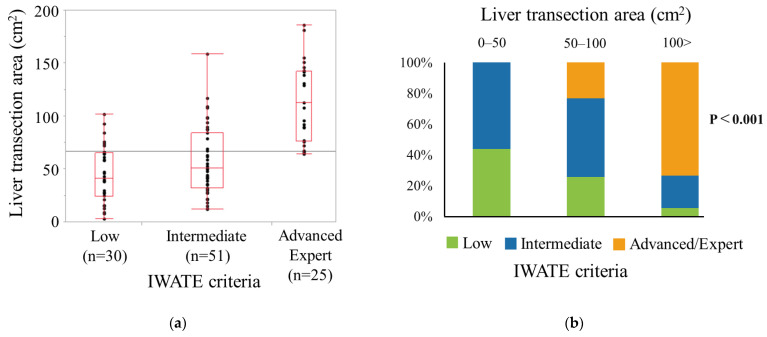
(**a**) The association between the liver transection area and difficulty level evaluated by the IWATE Criteria. A box-and-whiskers plot showing minimum-to-maximum liver transfection results across different surgical difficulty classes. (**b**) The proportion of difficulty levels stratified by the liver transection area category (0–50, 50–100, and >100).

The results of the investigation of the relationship between the LTA with the IWATE Criteria are shown in Figure 2. The median LTA was 41.5 (IQR, 24.0–65.3) cm^2^ for the low difficulty level group (*n* = 30), 51.0 (IQR, 32.0–84.0) cm^2^ for the intermediate difficulty level group (*n* = 51), and 113.0 (IQR, 76.5–142.5) cm^2^ for the advanced and expert difficulty level group (*n* = 25). The LTA was significantly associated with surgical difficulty, as evaluated using the IWATE Criteria. As shown in Figure 2b, some of the findings indicated that a low difficulty level required a larger LTA, >100 cm^2^.

### 3.3. Association between Liver Transection Area and Operative Time

**Figure 3:**
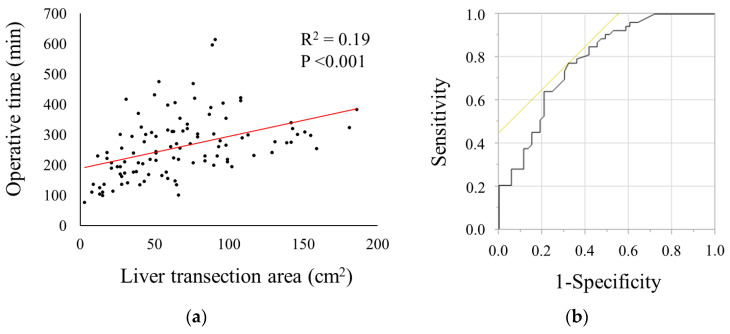
(**a**) The relationship between liver transection area and operative time; (**b**) the receiver operating characteristic curve showing the cutoff value of the liver dissection area for a prolonged operative time (cutoff value, 59.0 cm^2^; area under the curve, 0.78).

Figure 3a shows the relationship between the LTA and the operative time. The LTA was significantly correlated with the operative time (r^2^ = 0.19, *p* < 0.001). The receiver operating characteristic curve revealed that the cutoff value of the LTA for a prolonged operative time (>250 min) was 59.0 cm^2^ (area under the curve = 0.78) (Figure 3b).

### 3.4. Predictive Factor Associated with Prolonged Operative Time

Table 2 shows the results of the univariate and multivariate analyses that were deployed to investigate the predictive factors related to a prolonged operative time (>250 min). In the univariate analysis, five variables were found to be significant factors: primary disease (hepatocellular carcinoma), the tumor size (≥3 cm), the Iwate location score (≥4), the LTA (≥59 cm^2^), and the type of LLS (≥segmentectomy).

The multivariable analyses found that primary disease (hepatocellular carcinoma) (OR, 3.05; 95% CI, 1.17–8.36; *p* = 0.02), the LTA (≥59 cm^2^) (OR, 6.07; 95% CI, 2.38–16.6; *p* < 0.001), and the type of LLS (≥segmentectomy) (OR, 3.79; 95% CI, 1.35–11.4; *p* = 0.01) were significant factors associated with a prolonged operative time.

### 3.5. The Model Performance and Calibration of the Model

**Figure 4:**
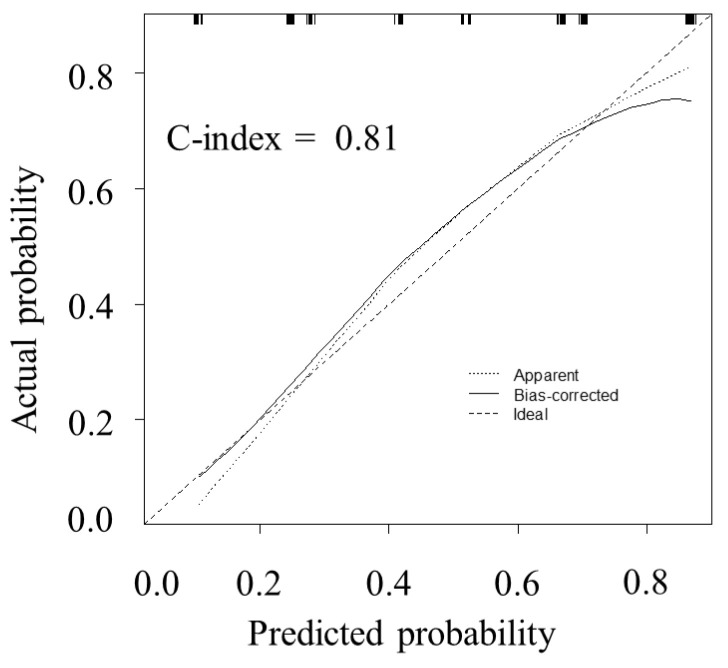
The calibration plot of the model for predicting a prolonged operative time.

Using independent factors associated with a prolonged operative time (primary disease, the LTA, and the hepatectomy type), internal validation was performed using the bootstrap method. The calibration plots of the predictive model for a prolonged operative time (>250 min) are shown in Figure 4. The probability of a prolonged operative time highly correlated with the actual probability, although it slightly underestimated the incidence in the middle-risk group. The C-index of the model was 0.81.

## 4. Discussion

To the best of our knowledge, this is the first study to investigate the effect of the LTA on surgical difficulty in LLS. We found a significant association between the LTA and the difficulty scoring system and a correlation between the LTA and operative time. Moreover, multivariate analyses suggested that the LTA was a significant parameter for predicting surgical difficulty in patients with LLS.

Several difficulty scoring systems have been developed, including the Ban [5], Iwate [6], Institut Mutualiste Montsouris [13], and Southampton difficulty scoring systems [14]. The usefulness of these scoring systems was also examined [7]. The variables of the previous difficulty scoring systems for LLS included the resection extent, tumor location, tumor size, liver function, preoperative chemotherapy, and previous open liver surgery [7]. However, conventional LLS difficulty scoring systems do not consider the LTA as a parameter [5,6]. Although previously suggested factors, including the tumor location, tumor size, and extent of liver resection, are important parameters, surgical difficulty should differ depending on the extent of the LTA, even within the same hepatic resection (Figure 1).

An analysis of the association between the LTA and difficulty level revealed that the LTA was significantly associated with surgical difficulty (Figure 2). In this study, we used the operative time as a predictor of surgical difficulty in LLS because it is a commonly used parameter that reflects the difficulty of LLS [7]. Using the cutoff value of the LTA for a prolonged operative time, we found that the LTA (≥59 cm^2^) was an independent factor for predicting surgical difficulty (Table 2). Our multivariate analyses revealed that the extent of resection was also a significant predictor of surgical difficulty. However, the tumor location and size were not significantly associated with surgical difficulty in LLS. Internal validity was assessed using a multivariate analysis (Figure 4), and the calibration plots of the model showed excellent agreement, with a C-index of 0.81. The LTA can be easily measured using preoperative CT images. Therefore, we believe that the LTA is a helpful index for estimating surgical difficulty in LLS.

This study had several limitations, including its small sample size and single-center retrospective nature, that should be considered when interpreting our findings. Regarding the predictive factors for surgical difficulty in LLS, unknown or residual confounding factors may be associated with a prolonged operative time. Although the extent of liver resection was determined by considering oncological factors and liver function in dedicated multidisciplinary meetings, the extent of the LTA may have been influenced by the surgeon beyond the principles of oncologic radicality. Moreover, while surgical liver transection lines were confirmed to correspond to those predicted preoperatively by the Vincent system, they may have been influenced by several factors, such as the quality of the liver parenchyma and intraoperative bleeding. Finally, the significance of the LTA should be confirmed in future studies with larger patient cohorts.

## 5. Conclusions

This study investigated the effect of the LTA on surgical difficulty in patients with LLS. The LTA was significantly associated with the surgical level evaluated using the IWATE Criteria as well as the operative time in LLS. The LTA is a useful parameter that reflects the difficulty of LLS.

## Figures and Tables

**Table 1:** Patient characteristics.

Variable	*n* = 106
Age, median, years	68 (59–73)
Sex (men/women), *n* (%)	63 (59.4)/43 (40.6)
BMI, median, kg/m^2^	23.4 (20.7–25.9)
ASA (1–2/3), *n* (%)	98 (92.5)/8 (7.5)
Comorbidity, *n* (%)	
Diabetes	20 (18.9)
Hypertension	35 (33.0)
HBV positive	32 (30.2)
HCV positive	12 (11.3)
Previous abdominal surgery, *n* (%)	46 (43.4)
Liver function	
Albumin, median, g/dL	4.1 (3.8–4.3)
Platelet, median, ×10^4^/μL	21 (15.8–25.9)
Prothrombin time, median, %	101 (94–110)
ICG-R15, median, %	9.4 (5.9–14.1)
Child–Pugh score (A/B), *n* (%)	106 (100)/0 (0)
Primary disease, *n* (%)	
Hepatocellular carcinoma	45 (42.5)
Intrahepatic cholangiocarcinoma	5 (4.7)
Metastatic tumor	47 (44.3)
Benign tumor	9 (8.5)
Tumor factor	
Tumor size, median, mm	20 (12–40)
Tumor number (solitary/multiple), *n* (%)	83 (78.3)/23 (21.7)
Iwate Difficulty score, *n* (%)	
Low (1–3)	30 (28.3)
Intermediate (4–6)	51 (48.1)
Advanced (7–9)	18 (17.0)
Expert (10–12)	7 (6.6)
VINCENT simulation	
Liver transection area, median, cm^2^	62.5 (36.0–91.8)
Operative factor	
Type of hepatectomy, *n* (%)	
Partial resection	54 (50.9)
Left lateral sectionectomy	19 (17.9)
Segmentectomy	3 (2.8)
Sectionectomy (except lateral sectionectomy)	18 (17.0)
Hemihepatectomy	12 (11.3)
Operative time, median, min	250 (195–310)
Blood loss, median, mL	65 (10–170)
Postoperative factor	
Mortality, *n* (%)	0 (0)
Major complications (CDc ≥ 3), *n* (%)	4 (3.8)
Hospital stay, median, day	8 (7–10)

BMI, body mass index; ASA, American Society of Anesthesiologists; HBV, hepatitis B virus; HCV, hepatitis C virus; ICG, indocyanine green; CDc, Clavien–Dindo classification.

**Table 2:** Univariate and multivariable analyses of factors associated with prolonged operative time (>250 min).

Variables	Univariate Analysis			Multivariable Analysis		
	OR	95% CI	*p* Value	OR	95% CI	*p* Value
Age (years)						
≥70 (vs. <70)	0.73	0.33–1.59	0.43			
Gender						
Male (vs. Female)	1.48	0.68–3.26	0.32			
BMI (kg/m^2^)						
≥25 (vs. <25)	1.47	0.64–3.45	0.36			
ASA						
3 (vs. 1–2)	0.58	0.11–2.48	0.46			
Previous abdominal surgery						
Presence (vs. absence)	1	0.46–2.16	1			
Primary disease						
Hepatocellular carcinoma (vs. others)	3.37	1.51–7.78	0.003	3.05	1.17–8.36	0.02
Tumor size (cm)						
≥3 (vs. <3)	2.26	1.02–5.14	0.04	0.95	0.30–2.83	0.92
Iwate location score						
≥4 (vs. <4)	2.42	1.06–5.77	0.04	2.25	0.80–6.71	0.13
Liver transection area (cm^2^)						
≥59 (vs. <59)	7.24	3.13–17.8	<0.001	6.07	2.38–16.6	<0.001
Type of hepatectomy						
≥Sectionectomy (vs. <Sectionectomy)	3.82	1.73–8.74	<0.001	3.79	1.35–11.4	0.01

BMI, body mass index; ASA, American Society of Anesthesiologists; OR, odds ratio; CI, confidence interval.

## Data Availability

The original contributions presented in the study are included in the article, further inquiries can be directed to the corresponding author.
